# Cerebrospinal Fluid Pressure-Related Features in Chronic Headache: A Prospective Study and Potential Diagnostic Implications

**DOI:** 10.3389/fneur.2018.01090

**Published:** 2018-12-18

**Authors:** Francesco Bono, Maria Curcio, Laura Rapisarda, Basilio Vescio, Caterina Bombardieri, Domenica Mangialavori, Umberto Aguglia, Aldo Quattrone

**Affiliations:** ^1^Center for Headache and Intracranial Pressure Disorders, Magna Græcia University, Catanzaro, Italy; ^2^Institute of Neurology, Department of Medical and Surgical Sciences, Magna Græcia University, Catanzaro, Italy; ^3^Neurosciences Research Center, Magna Græcia University, Catanzaro, Italy; ^4^Institute of Neuroradiology, Department of Medical and Surgical Sciences, Magna Græcia University, Catanzaro, Italy; ^5^Institute of Ophthalmology, Department of Medical and Surgical Sciences, Magna Græcia University, Catanzaro, Italy

**Keywords:** isolated CSF hypertension, intracranial hypertension, one-hour lumbar CSF pressure monitoring via a spinal puncture needle, CSF pressure pulsations, chronic headache

## Abstract

**Objective:** To identify the pressure-related features of isolated cerebrospinal fluid hypertension (ICH) in order to differentiate headache sufferers with ICH from those with primary headache disorder.

**Methods:** In this prospective study, patients with refractory chronic headaches and suspected of having cerebrospinal fluid-pressure elevation without papilledema or sixth nerve palsy, together with controls, underwent 1-h lumbar cerebrospinal fluid pressure monitoring via a spinal puncture needle.

**Results:** We recruited 148 consecutive headache patients and 16 controls. Lumbar cerebrospinal fluid pressure monitoring showed high pressure and abnormal pressure pulsations in 93 (63 %) patients with headache: 37 of these patients with the most abnormal pressure parameters (opening pressure above 250 mm H_2_O, mean pressure 301 mm H_2_O, mean peak pressure 398 mm H_2_O, and severe abnormal pressure pulsations) had the most severe headaches and associated symptoms (nocturnal headache, postural headache, transient visual obscuration); 56 patients with the less abnormal pressure parameters (opening pressure between 200 and 250 mm H_2_O, mean pressure 228 mm H_2_O, mean peak pressure 316 mm H_2_O, and abnormal pressure pulsations) had less severe headaches and associated symptoms.

**Conclusions:** Nocturnal and postural headache, and abnormal pressure pulsations are the more common pressure-related features of ICH in patients with chronic headache. Abnormal pressure pulsations may be considered a marker of ICH in chronic headache.

## Introduction

Isolated cerebrospinal fluid (CSF) hypertension refers to the occurrence of CSF-pressure elevation in the cranial-spinal compartment without an identifiable cause. As we investigated the abnormalities of CSF pressure in patients with chronic headache by using a novel method of lumbar CSF pressure monitoring, and the pressure parameters evaluated for diagnosing elevated CSF pressure in the cranial-spinal compartment are not included in the ICHD-3 diagnostic criteria of idiopathic intracranial hypertension (1), the term of isolated CSF hypertension (ICH) is used to name a condition of elevated CSF pressure in the cranial-spinal compartment rather than idiopathic intracranial hypertension or pseudotumor cerebri.

ICH manifests with symptoms and signs such as papilledema and sixth nerve palsy. However, such symptoms may be also lacking in patients with high CSF pressure (2, 3). In such cases headache is the most common clinical manifestation of ICH (4, 5). However, recognizing that a headache is due to ICH is difficult because the headache profile of patients with ICH does not differ from that of patients with primary headache disorder (6–10).

CSF pressure measurement creates further confusion. The technique of continuous recording of CSF pressure found that patients with intracranial hypertension due to brain tumor or hydrocephalus had rapid large pulsations in pressure in form of B waves and plateau waves. Whereas, small pulsations in CSF pressure synchronous with respiration (ranging 10–30 mm CSF) and with systole (ranging 20–30 mm CSF) were observed in normal subjects (11). It is now recognized that headache sufferers without papilledema or sixth nerve palsy but with a normal or slightly elevated CSF opening pressure during a single-spot CSF pressure measurement may manifest high mean CSF pressure and abnormal pressure pulsations in form of B waves (ranging 65–680 mm CSF) when undergoing CSF pressure monitoring (12–14).

Given the difficulty of diagnosing headache attributable to isolated high CSF pressure, and the fact that a single-spot CSF opening pressure measurement may be insufficient to identify high CSF pressure in headache sufferers, recognition of headache attributable to high CSF pressure in the absence of papilledema or sixth nerve palsy is subject to much debate in clinical practice (15). As a result, headache sufferers with isolated high CSF pressure may experience considerable delay in their diagnosis and treatment.

This highlights a need to identify the characteristics and the pressure-related features of ICH in patients with chronic headache who had no evidence of papilledema and sixth nerve palsy in order to differentiate headache sufferers with ICH from those with primary headache disorder. In this study, our focus was therefore on headache sufferers suspected of having isolated high CSF pressure.

## Methods

Institutional Review Board approval was obtained for this prospective, single-center, cohort study, and written informed consent was obtained from all the participants.

### Patient Selection

Screening of headache sufferers with refractory chronic headaches and associated symptoms (nocturnal or postural headache, intracranial noises, visual disturbances, pulsatile tinnitus, and vertigo) who were suspected of having high CSF pressure, and patients with non-organic neurologic disorder (psychogenic movement disorders or psychiatric diseases) who had undergone a lumbar puncture for a reason other than chronic headache was conducted between December 2012 and February 2018 at the referral center for Headache and Intracranial Pressure Disorders of the Institute of Neurology in Catanzaro. Patients were enrolled in the study if they met the following inclusion criteria: absence of papilledema (confirmed by a complete ophthalmologic evaluation) and of sixth nerve palsy; normal gadolinium enhanced MR of the brain; no history of systemic disease or drug intake known to be associated with elevated CSF pressure; and no evidence of current or prior cerebral venous thrombosis. The criteria for exclusion from the study were: abnormal neurological examination; MR evidence of structural brain lesions; cerebral venous thrombosis; hydrocephalus; and arterial hypertension.

### Assessment

Participants were asked to complete standardized forms so as to provide us with details of their general medical history (including body mass index: weight in kilograms divided by the square of height in meters), and of their neurological, ophthalmological, and family histories. All patients were interviewed and examined by the same two neurologists, and headaches were diagnosed according to the criteria established by the IHS (1). Postural headache were diagnosed whether headache worse in recumbence and improved in the upright posture. To avoid risk of diagnostic errors resulting from differing diagnoses by the two neurologists, the principal investigator (F. B.) evaluated the patient in debated cases.

The participants underwent magnetic resonance (MR) of the brain and a cerebral MR venography (MRV) before lumbar puncture (LP). They underwent LP so that CSF opening pressure could be measured and CSF pressure could be monitored for 1 h via a spinal puncture needle.

The validated 0–10 visual analog scale (VAS) and the Migraine Disability Assessment (MIDAS) questionnaire were administered both at baseline and during the follow-up for assessing pain and disability. Higher scores on VAS and MIDAS served to indicate the severity of headache. Mood and anxiety were assessed with Beck Depression Inventory-II (BDI-II), and Hamilton Anxiety Rating Scale (HARS) at baseline and during the follow-up. Besides, we asked patients to fill in a headache diary throughout the study. A respondent to medical treatment (good outcome) was considered a subject who achieved a 50% or greater reduction in number of headache days per month.

ICH was diagnosed if the patient met the following diagnostic criteria: (i) at least two or more of the following symptoms of raised CSF pressure: postural or nocturnal headaches, headache aggravated by coughing or worse on awakening, transient visual obscurations, photopsias, vertigo, pulsatile tinnitus, intracranial noises; (ii) at least two or more of the following neuroimaging signs: bilateral transverse sinus stenosis, empty sella, posterior sclera flattening, perioptic subaracnoid space distension; vertical tortuosity of the orbital optic nerve, tonsillar ectopia; (iii) altered pressure parameters for stage 1 or stage 2 of CSF hypertension; (iv) normal CSF contents; (v) symptoms relieved by a reduction in CSF pressure.

### CSF Pressure Measurement

The procedure outlined below has also been described in a previous report (12). The same operator (FB) performed the LP and the 1-h lumbar CSF pressure monitoring via a spinal puncture needle on each patient. Pre-treatment of patients was done via an intramuscular antibiotic agent (ceftriaxone), oral lorazepam (0.5 mg), and local anesthesia (lidocaine). With the subject placed in the left lateral decubitus position in a puncture room, a standard 20-gauge (occasionally 22-gauge) Quincke pointed spinal needle with a three-way stopcock was inserted at L_3_-_4_ or L_4_-_5_ interspaces. The pressure transducer (Transducer kit, Edwards Lifesciences, Irvine, CA, USA) was attached to the hub of needle via a 10-cm long flexible tube without loss of CSF. It was placed at the level of the same horizontal plane as the estimated level of the spinal puncture needle in the subarachnoid space. It was then zeroed. A pressure cable linked the transducer to the monitor (Passport V, Datascope Corporation, Mahwah, NJ, USA). At the start, opening pressure was recorded for 4 min to be certain that the pressure values had stabilized. Immediately after, CSF pressure was monitored for a 60-min period in order to record the mean pressure, highest peak pressure, mean pulse amplitude, and abnormal fluctuations in pressure. Physician observation and blood pressure evaluation were continuous in order to analyze CSF pressure only during artifact-free epochs, and to exclude arterial hypertension. At the end of the procedure the closing pressure was measured. A CSF removal (about 15 ml) was performed only in patients with altered CSF pressure parameters.

Normal CSF pulsations were considered rapid changes in pressure synchronous with respiration and with systole ranging between 10 to 30 mm CSF. Abnormal pressure pulsations in the form of B waves were identified as repetitive pulsations in CSF pressure occurring one to six times per minute in periods of more than 10 min with pressure elevations from 65 mm H_2_O to 680 mm H_2_O (16). B waves indeed are associated with rhythmic alterations of cerebral blood flow, they may occur in brain tumor, hydrocephalus and idiopathic intracranial hypertension (11).

### Image Analysis

MR and cerebral MR venographies of the brain were performed on all subjects, using a 1.5 T scanner (GE Medical Systems, Milwaukee, WI). Brain MR consisted of sagittal T1- and transverse T2-weighted spin echo sequences with a slice thickness of 5 mm and an interslice gap of 1 mm. MRV was performed using 3D-PC techniques as described elsewhere (17). All brain MR and MR venographies were analyzed by the same neuroradiologist, who were blinded to each patient's history. We classified the transverse sinus (TS) stenosis (TSS) as present or absent. TSS was considered present when the signal flow was poor or lacking (flow gap) in the mid-lateral portion of the transverse sinus (unilateral TSS) or in both transverse sinuses (BTSS).

### Statistical Analyses

Differences in sex distribution and in the distribution of binary variables have been assessed by means of the Fisher's exact test, followed by pairwise proportion test. The Shapiro-Wilk test was used to check for normality before performing comparisons. The Kruskal-Wallis test, followed by the pairwise Wilcoxon rank sum test, was used to assess differences in age at examination, opening pressure, pulse amplitude, BDI-II and HARS and MIDAS questionnaire between groups. Body-Mass Index, mean pressure, and highest peak pressure were compared using the ANOVA test followed by the pairwise *t*-test. All *p*-values were corrected according to Bonferroni. Logistic regression models were used to assess linear relationships linking the odds of symptoms to pulse amplitude and to the presence of pressure pulsations. Models were chosen according to minimum Akaike Information Criterion. Statistical analysis was performed with R Statistical software (R for Unix/Linux, version 3.1.1, the R Foundation for Statistical Computing, 2014).

## Results

### Participants

Participant characteristics are set out in Table 1. We enrolled 101 patients with chronic migraine (CM), 47 patients with chronic tension-type headache (CTTH), and 16 patients with non-organic neurologic disorder who had undergone a lumbar puncture for a reason other than chronic headache.

**Table 1:** Demographic and characteristics of 148 patients with chronic headache.

Age, y, mean ± SD	41.8 ± 14.4
Sex, F/M	129/19
Body mass index, kg/m^2^, mean ± SD	30.5 ± 6.2
Headache duration, y, mean ± SD	8.2 ± 1.1
**Headache diagnoses**, ***n* (%)**	
Chronic migraine	101 (68)
Chronic tension-type headache	47 (32)
**Headache profile**, ***n* (%)**	
Unilateral head pain	56 (38)
Diffuse head pain	92 (62)
Pulsating pain	90 (61)
Moderate	46 (31)
Severe	104 (69)
Daily	86 (58)
Worse in the early morning and aggravated after coughing	65 (44)
Nocturnal head pain attacks	59 (40)
Positional headache	78 (53)
Overuse medication, *n* (%)	56 (38)
Beck Depression Inventory-II, mean ± SD	12.4 ± 9.7
Hamilton Anxiety Scale, mean ± SD	16.1 ± 9.1
Migraine Disability Assessment, mean ± SD	27.1 ± 5.3
Visual Analog Scale, mean ± SD	8 ± 1
**Associated symptoms**, ***n* (%)**	
Visual disturbances	37 (25)
Pulsatile tinnitus	60 (41)
Vertigo	35 (24)
Intracranial noises and other	37 (25)

### CSF Pressure Findings

Of the 148 patients, 93 had abnormal CSF pressure pulsations, with opening pressures ranging from 206 to 385 mm H_2_O, highest peak pressure above 270 mm H_2_O, and mean pulse amplitude >65 mm H_2_O. The remaining 55 patients and 16 controls had both normal pulsations in CSF pressure, with both opening pressures and mean CSF pressures below 200 mm H_2_O (Table 2).

**Table 2:** Characteristics of patients with chronic headache and controls grouped according to CSF opening pressure findings.

**Characteristics**	**Group 1**	**Group 2**	**Group 3**	**Controls**	***p*-value**
	**Opening pressure < 200 mm H_**2**_O *n* = 55**	**Opening pressure 200-250 mm H_**2**_O *n* = 56**	**Opening pressure >250 mm H_**2**_O *n* = 37**	**Opening pressure < 200 mm H_**2**_O *n* = 16**	
Age; mean ± SD^*^, ^*a*^	44.7 ± 12.5	38.8 ± 14	42.1 ± 16.8	38.1 ± 14.8	0.15
Sex: M/F^@^, ^a^	6/49	10/46	3/34	5/11	0.38
BMI, kg/m^2^, mean ± SD^#^, ^a^	27.6 ± 5.7	32 ± 6.2	33.1 ± 5	27.5 ± 5.2	< 0.001
**CSF pressure measurement, mean ± SD**					
Opening pressure^*^, ^*a*^	138.7 ± 28.1	231.8 ± 12.4	282.2 ± 32.0	157.9 ± 27.1	< 0.001
Mean pressure^#^, ^a^	154.3 ± 26	228.3 ± 15.5	301 ± 36.9	164.7 ± 21.1	< 0.001
Highest Peak pressure^#^, ^a^	196.9 ± 32.1	316.2 ± 45.8	398.1 ± 60.7	198.3 ± 26.2	< 0.001
Pulse amplitude^*^, ^*a*^	24.4 ± 15.7	95.0 ± 34.7	143.0 ± 55.6	45.5 ± 11.0	< 0.001
Pressure pulsations, *n* (%)^@^, ^b^	1 (2)	55 (100)	37 (100)	n.a.	< 0.001
**Headache diagnosis**, ***n*** **(%)**^**@**^, ^**b**^					
Tension-type headache	14 (25)	23 (41)	10 (27)	n.a.	0.17
Migraine headache	41 (75)	33 (59)	27 (73)	n.a.	0.17
Pre-existing primary headache	0	52 (93)	35 (95)	n.a.	< 0.001
**Headache profile**, ***n*** **(%)**^**@**^, ^**b**^					
Diffuse head pain	31 (55)	39 (70)	22 (59)	n.a.	0.32
Pulsating pain	19 (36)	33 (59)	28 (76)	n.a.	< 0.001
Severe	22 (40)	46 (79)	36 (97)	n.a.	< 0.001
Daily	15 (27)	36 (64)	35 (95)	n.a.	< 0.001
Aggravated with coughing	5 (9)	29 (52)	31 (84)	n.a.	< 0.001
Nocturnal head pain attacks	4 (7)	27 (48)	28 (76)	n.a.	< 0.001
Positional headache	2 (4)	43 (77)	33 (89)	n.a.	< 0.001
**Associated symptoms**, ***n*** **(%)**^**@**^, ^**b**^					
Pulsatile tinnitus	11 (20)	27 (48)	22 (59)	n.a.	< 0.001
Visual disturbances	1 (2)	13 (23)	23 (62)	n.a.	< 0.001
Vertigo	8 (14)	16 (29)	11 (30)	n.a.	0.14
Intracranial noises	2 (4)	15 (27)	19 (51)	n.a.	< 0.001
**Non-organic neurological disorders**					
Psychogenic movement disorder	n.a.	n.a.	n.a.	12	
Psychiatric disease	n.a.	n.a.	n.a.	4	
**Neuroimaging findings**, ***n*** **(%)**^**@**^, ^**b**^					
Empty sella	4 (7)	32 (57)	25 (68)	n.a.	< 0.001
Perioptic subaracnoid space distension	1 (2)	15 (27)	21 (57)	n.a.	< 0.001
Bilateral TSS	1 (2)	40 (71)	29 (78)	n.a.	< 0.001
Unilateral TSS	15 (27)	6 (11)	6 (16)	4 (25)	0.08
Normal-appearing TS	39 (71)	10 (18)	2 (6)	12 (75)	< 0.001

*BMI, body mass index; CSF, cerebrospinal fluid; TS, transverse sinus; TSS, transverse sinus stenosis; n.a., not applicable. Data are given as mean ± SD*.

* *Kruskal-Wallis test, followed by pairwise Wilcoxon rank sum test*.

@ *Fisher's exact test, followed by pairwise proportion test*.

# *ANOVA test, followed by pairwise t-test*.

a *p-value referred to comparison between Group 1, Group 2, Group 3, and Controls*.

b *p-value referred to comparison between Group 1, Group 2 and Group 3. Post-hoc analyses are reported in Supplementary Tables 1, 2. All p-values corrected according to Bonferroni*.

**Figure 1:**
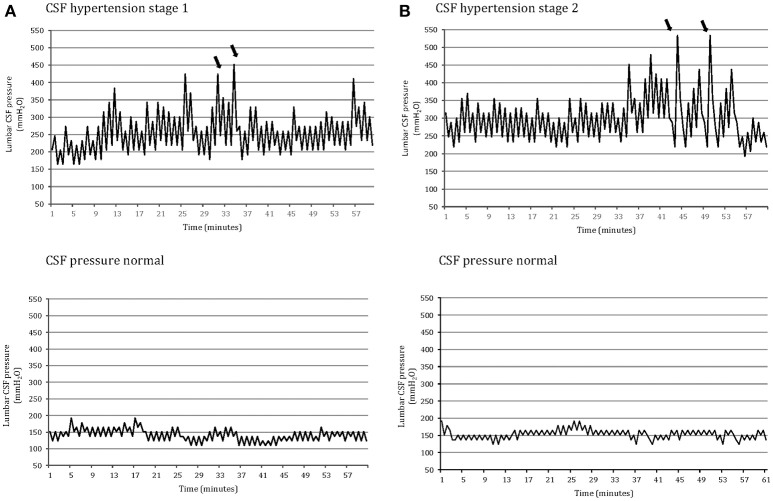
One-hour lumbar cerebrospinal fluid (CSF) pressure monitoring via a spinal puncture needle: abnormal pressure pulsations (B waves) and highest peak pressure (arrow) in a patients with opening pressure between 200 and 250 mm H_2_O associated with moderate/severe chronic headache and tinnitus (CSF hypertension: stage 1); severe pressure pulsations (B waves) (arrow) and highest peak pressure in a patient with opening CSF pressure above 250 mm H_2_O associated with severe chronic headache and visual disturbances (CSF hypertension: stage 2); normal pressure pulsations associated with opening pressure below 200 mm H_2_O in both a patient with primary chronic headache **(A)** and a control subject without headache **(B)** (CSF pressure: normal).

On the basis of these CSF pressure findings (opening pressure, highest peak pressure, mean pulse amplitude, the presence and severity of abnormal pressure pulsations), patients were divided into three groups (Table 3). Group 1 comprised 55 patients, each with an opening pressure below 200 mm H_2_O (mean = 138.7 mm H_2_O, *SD* = 28.1; range 82–192), normal mean CSF pressure (154.3 mm H_2_O, *SD* = 26) and with no evidence of abnormal pressure pulsations (Figure 1). Group 2 comprised 56 patients, each with a slight elevation of CSF opening pressure (mean = 231.8 mm H_2_O, *SD* = 12; range 206–247), elevated mean pressure (228.3 mm H_2_O, *SD* = 15) associated with abnormal pressure pulsations (Figure 1). Group 3 included 37 patients, all with both elevated CSF opening pressure (mean = 282.2 mm H_2_O, *SD* = 32, range 261–385), elevated mean CSF pressure (mean = 301 mm H_2_O, *SD* = 36) associated with severe abnormal pressure pulsations (Figure 1).

**Table 3:** Classification of CSF pressure findings in 148 patients with chronic headache and 16 controls.

**Category**	**CSF pressure *mm H*_2_*O***
Normal *Isolated chronic headache* *or non-organic neurologic disorder*	<200 opening pressure <200 mean pressure <65 mean pulse amplitude <270 highest peak pressure Normal pressure pulsations
CSF Hypertension* Stage 1 *Moderate/severe chronic headache, Associated symptoms*	200–250 opening pressure 200–250 mean pressure >65 mean pulse amplitude >270 highest peak pressure Moderate abnormal pressure pulsations >250 opening pressure
Stage 2 *Severe chronic headache,* *Associated symptoms*	>250 mean pressure; >65 mean pulse amplitude; >300 highest peak pressure; Severe abnormal pressure pulsations

CSF denotes cerebrospinal fluid; *CSF hypertension is defined as an opening pressure above 200 or greater, mean pressure above 200, >65 mean pulse amplitude, >270 peak pressure, presence of abnormal pressure pulsations. The stage is determined on the basis of the opening pressure, and of the results of the average of 60 min of continuous CSF pressure recording through lumbar puncture needle.

### CSF Pressure-Related Features

The headache profile and the associated symptoms of the patients with abnormal CSF pressure parameters differed from those of patients who had normal CSF pressure parameters (Table 2). There was a strong relationship between pulse amplitude, the presence of abnormal pressure pulsations and symptoms (Supplementary Tables 1–3). In Group 3, patients with severe abnormal pressure pulsations and mean pressure >250 mm H_2_O had the most severe headaches (highest scores on VAS and MIDAS) and associated symptoms (high frequency of: nocturnal headache, postural headache, photopsias, transient visual obscurations). In Group 2, patients with less severe abnormal pressure pulsations and mean pressure >200 mm H_2_O, had less severe symptoms; in Group 1, patients who had no abnormal pressure pulsations and mean pressures below 200 mm H_2_O had primary chronic headache disorder (Figure 1). Postural headache and nocturnal headache were the more common symptoms of patients with the most abnormal CSF pressure parameters, and also the body mass index was significantly different among the groups (Table 2). All of the subjects in the control group, all with opening pressures below 200 mm H_2_o and normal CSF pressure pulsations, had no headache.

### Brain MRI and Cerebral MR Venography Findings

**Figure 2:**
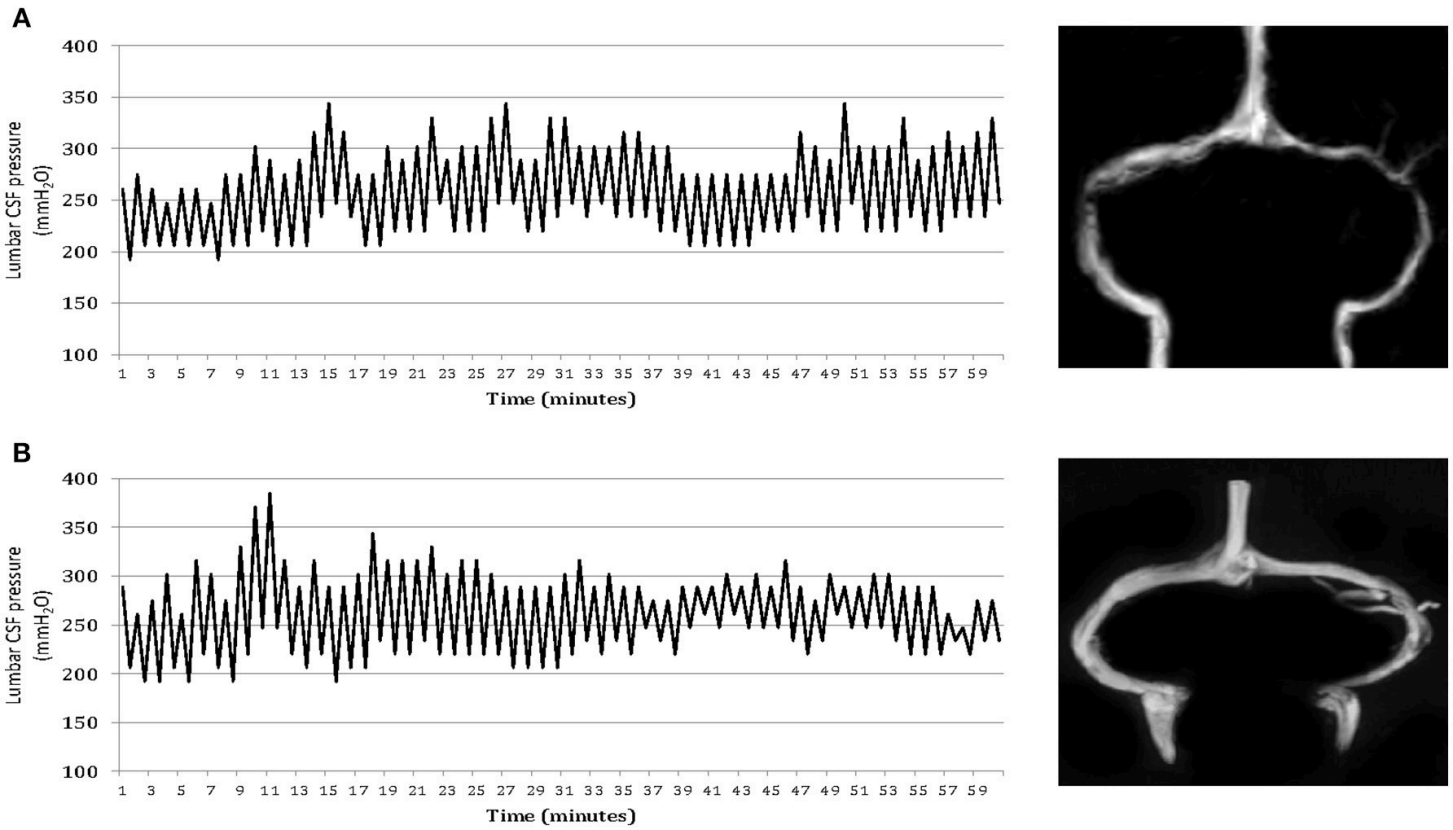
One-hour lumbar cerebrospinal fluid (CSF) pressure monitoring via a spinal puncture needle. Elevated mean CSF pressures and abnormal pressure pulsations in both a patient with isolated CSF hypertension (ICH) associated with bilateral transverse sinus stenosis **(A)** and a patient with ICH associated with normal appearance of the transverse sinuses **(B)**.

Brain MRI findings of the participants are summarized in Table 2. Forty patients (71%) in Group 2 and 29 patients (78%) in Group 3 displayed marked stenosis in the mid-lateral portion of both TS, whereas 54 patients in Group 1 had normal appearance of TS or unilateral TS stenosis on cerebral MRV. Moreover, 24 (22%) of headache patients with abnormal CSF pressure pulsations and high CSF pressure had normal appearance of TS or unilateral TSS (Figure 2). Whereas, controls had no abnormal pressure pulsations with pressures below 200 mm H_2_O and normal appearance of TS or unilateral TSS on MRV (Table 2).

### Outcomes

**Figure 3:**
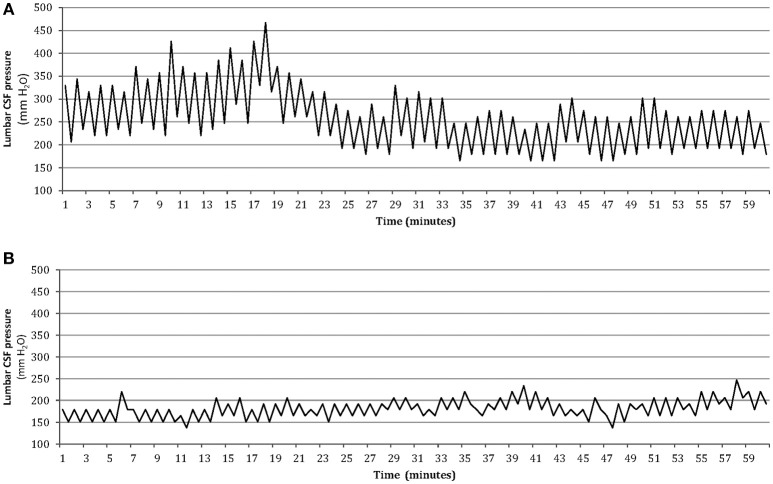
One-hour lumbar CSF pressure monitoring via a spinal puncture needle. Abnormal pressure pulsations associated with elevated mean CSF pressure in the patient at baseline **(A)**, and disappearance of abnormal pressure pulsations with normal mean pressure after medical treatment in the same patient during the follow-up **(B)**.

An improvement in the intensity of pain and frequency of headaches was observed following CSF removal in all patients with ICH. There was a lower score on VAS (3 ± 2) and a reduction in number of headache days (mean reduction 67%). It should be noted that none of the patients with ICH developed postural headache after LP or reported a worsening of headache during follow-up. We recommended an informal weight reduction diet program in all obese patients and prescribed acetazolamide (500–1,000 mg/day) and topiramate (50–100 mg/day) simultaneously for the majority of patients with ICH. The rationale for using topiramate in this condition is its broad mechanistic profile (including an inhibitor effect on carbonic anhydrase isoenzymes II and IV). Hence, the simultaneous administration of topiramate and acetazolamide may have a synergic effect on headache and high CSF pressure in these patients (12). Patients were instructed to address questions concerning the medical treatment to physician throughout the study, and the ability of the patients to tolerate the treatment was satisfactory. The response to combined treatment with topiramate and acetazolamide was good in most patients with ICH, and 69 (74%) out 93 patients achieved ≥50% reduction in headache days. During the follow-up, five of these patients agreed to undergo a second lumbar CSF pressure monitoring. This showed normal pulsations in pressure and a decreased average pressure (Figure 3).

### Adverse Events

To improve patient compliance during the lumbar CSF pressure monitoring, local anesthesia and a mild sedation were given before LP. This avoided confounding factors such as patient pain and anxiety and pressure artifacts during continuous pressure recording. Nine (6%) hence complained of discomfort during the procedure. While there were no major adverse events associated with lumbar CSF pressure monitoring, 15 (27%) headache sufferers without altered CSF pressure parameters experienced temporary (2–3 h) discomfort and mild headache soon after the procedure.

## Discussion

Our results provide evidence that nocturnal and postural headache, and abnormal pressure pulsations are the most common pressure-related features of isolated high CSF pressure in patients with chronic headache. Moreover, abnormal pressure pulsations are the main pressure findings of ICH, and they may be considered a marker of ICH in patients with chronic headache who have no evidence of papilledema or sixth nerve palsy. In contrast, the lack of the abnormal pressure pulsations is associated with normal CSF pressure in both patients with primary chronic headache and non-headache controls. These findings suggest that abnormal CSF pressure pulsations may differentiate headache sufferers with high CSF pressure from those with primary headache disorder.

There was indeed an important difference in patients' clinical manifestations. Symptoms such as nocturnal attacks of pulsating pain and postural headache, vertigo, headache aggravated by coughing or pulsatile tinnitus were more common in patients with ICH than in headache patients without altered CSF pressure parameters. These differences may be explained by the occurrence of particularly abnormal pressure pulsations in patients with more severe symptoms. Indeed, nocturnal attacks of pulsating pain and postural headache were more common in patients with ICH. The abnormal pulsations in CSF pressure occurring during sleep may be the cause of nocturnal headache, while rapid pulsations in CSF pressure precipitated by posture changes may trigger the positional headache (18–21). This suggests that one possible basis for headache in these patients with ICH could be a sensitization of the intracranial meninges due to mechanical stimuli of recurrent abnormal CSF pressure pulsations. This sensitization of meningeal afferents to mechanical stimuli is recognized as the mechanism causing pulsating head pain aggravated by sudden head movements and coughing in patients with intracranial pathologies (22). The latter mechanism produces the peripheral sensitization leading to central sensitization, which in turn may contribute to the chronification of headache in these patients. It is worth noting that most patients with ICH had a chronic headache mimicking the pre-existing primary headache. To explain this mimicry, we hypothesize that abnormal pulsations in CSF pressure may trigger the same mechanism that cause the pain in a primary headache attack. The mimicry thereby emerges as a variation of pre-existing primary headache; as such, it could be considered a triggered headache rather than a secondary or primary one. This hypothesis is strengthened by evidence suggesting that primary and secondary headaches may share a similar biological mechanism (23–25). These observations, along with the data gathered, support our finding that clinical features are related to the presence and severity of abnormal pressure pulsations in headache sufferers with ICH.

Consistent with our results, 24-h intracranial pressure monitoring revealed abnormal pressure pulsations associated with high mean CSF pressure in 9 patients with idiopathic intracranial hypertension without papilledema (IIHWOP), even though in 5 of these patients the baseline CSF pressure was below 200 mm H_2_O (13). Moreover, 24-h lumbar CSF pressure monitoring via a catheter showed abnormal pressure pulsations in the form of B waves, plateau waves and near plateau waves in 10 patients with refractory chronic headache with IIHWOP, and 4 of these patients had CSF opening pressure below 250 mm H_2_O (14). In addition, 1-h lumbar CSF pressure monitoring through a spinal puncture needle demonstrated abnormal CSF pressure waves in headache sufferers with IIHWOP who had slightly abnormal opening pressure (12). Previous abovementioned studies emphasize that CSF-pressure elevation may be intermittent in patients with IIHWOP. They support our finding showing that abnormal pressure pulsations are associated with high CSF pressure in headache sufferers with no papilledema or sixth nerve palsy. The presence of such pulsations differentiates headache sufferers with ICH from those with normal CSF pressure.

The pathogenesis of these abnormal pulsations in CSF pressure (B waves and plateau waves) may be clarified by reference to the theory that CSF pressure is derived from the circulation of cerebral blood and cerebrospinal fluid (26). In fact, the disruption of the autoregulation of cerebral blood flow and poor compliance of cerebrospinal system may be both pathophysiological mechanisms causing the abnormal CSF homeostasis, which in turn leads to abnormal pressure pulsations and high CSF pressure (27). It bears emphasis that roughly two-thirds of headache sufferers with ICH had bilateral transverse sinus stenosis that caused cerebral venous outflow disturbances. B waves are associated with rhythmic alterations of cerebral blood flow, and plateau waves and near plateau waves are concomitant with an increase of cerebral blood volume (28). A reduced compliance of the spinal canal compartment, resulting in a delayed CSF absorption (29), might contribute to the development of high CSF pressure with abnormal pressure pulsations in the remaining headache sufferers who had no altered cerebral venous drainage. On this basis we speculate that the presence of the abnormal pulsations in pressure, indicating a failure of the compensatory mechanisms for maintaining a normal CSF pressure (11, 13, 26), signifies that CSF-pressure elevation is symptomatic. The presence of abnormal pulsations in CSF pressure may be considered a marker of high CSF pressure.

In the present study, use of 1-h lumbar CSF pressure monitoring via a spinal puncture needle enabled us to detect the symptomatic stage 1 of CSF hypertension in a portion of patients who had opening pressure between 200 and 250 mm H_2_O. Although a previous study reported an upper normal limit of 200 mm H_2_O for CSF opening pressure, irrespective of body mass index (30), an opening pressure of up to 250 mm H_2_O is now considered normal in adult subjects (31). This discrepancy may be explained by the fact that some authors (31) failed to consider the influence of sinovenous stenosis on the upper limit of normal CSF opening pressure (32, 33). As a result, the presence of sinovenous stenosis in their adult subjects with CSF pressure between 200 and 250 mm H_2_O cannot be excluded.

It is worth noting that on MRV some headache sufferers with stage 2 CSF hypertension (an opening and average pressure above 250 mm H_2_O with severe abnormal pressure pulsations) were found to present normal appearances of TS or unilateral TS stenosis. However, the presence of altered CSF pressure parameters during pressure monitoring was associated with symptoms of raised pressure, which were relieved by reducing CSF hypertension in these patients. This fact indicates a symptomatic elevation of CSF pressure, causing a chronic headache. Moreover, in the patients and controls with no abnormal pressure pulsations and with opening and average CSF pressures below 200 mm H_2_O, MRV revealed normal appearance of TS or unilateral TS stenosis. These data provide evidence for the first time that abnormal pressure pulsations and high CSF pressure may also occur without sinovenous stenosis.

Our results show that 1-h lumbar CSF pressure monitoring via a spinal puncture needle is a safe method of CSF pressure measurement, and also indicate that 60 min of pressure recording is sufficient to detect abnormal pressure pulsations when assessing headache sufferers suspected of having high CSF pressure. Continuous pressure recordings in these patients with ICH also demonstrated that CSF pressure may vary considerable over time. This suggests that CSF pressure assessment by measuring the height of the fluid column in the manometer line connected to a spinal puncture needle may be misleading in the case of headache sufferers. In everyday practice, lumbar CSF pressure monitoring via a spinal puncture needle for 60 min may identify either “false-positive” (34) (CSF pressure that is elevated when measured during a single-spot opening pressure measurement but that is otherwise normal) thereby preventing unnecessary treatment. It may also identify “false-negative” (12) (CSF pressure that, while otherwise elevated, reads as normal when measured during a single-spot opening pressure measurement) thereby indicating that treatment is necessary.

There are limitations to this study, in part linked to the fact that we investigated the CSF pressure-related features in patients with chronic headache by using a new method of lumbar CSF pressure measurement. The CSF pressure parameters considered for diagnosing isolated CSF hypertension in our patients with chronic headache are not included in the diagnostic criteria of idiopathic intracranial hypertension (1). We did not use the terminology of ICHD-3 in this study. We used the term of isolated CSF hypertension (ICH) to indicate stage 1 and stage 2 of altered CSF pressure in patients with chronic headache. Given the fact that lumbar CSF pressure monitoring lasted 1 h, only 16 patients with a non-organic neurologic disorder who had undergone a lumbar puncture for a reason other than chronic headache agreed to undergo continuous CSF pressure monitoring. In addition, in the current study we included candidates for lumbar CSF pressure measurement who had been admitted to referral center for headache and intracranial pressure disorders. This fact may explain why the percentage (25%) of patients with elevated CSF pressure proved higher than described in the literature.

Finally, our data demonstrate that the presence and the severity of abnormal pressure pulsations are related to clinical manifestations of ICH in patients with chronic headache. Our data highlight the effectiveness of lumbar CSF pressure monitoring via a spinal puncture needle over a 1-h period for detecting high CSF pressure in patients with chronic headache with no papilledema or sixth nerve palsy. They also suggest that CSF pressure is a continuous, not discrete, variable.

## Author Contributions

FB: study concept and design, acquisition, analysis, and interpretation of the data, drafting, revising the manuscript, including its theoretical contributions, final approval of the version to be published. MC, LR, CB, DM: acquisition of data and analysis of data, final approval of the version to be published. BV: statistical analysis, final approval of the version to be published. UA and AQ: revising the manuscript, final approval of the version to be published.

### Conflict of Interest Statement

The authors declare that the research was conducted in the absence of any commercial or financial relationships that could be construed as a potential conflict of interest.

## References

[1] Headache Classification Committee of the International Headache Society (IHS) The international classification of headache disorders, 3rd edition. Cephalalgia (2018) 38:1–211. 10.1177/0333102413485658 29368949

[2] Lipton HL Michelson PE Pseudotumor cerebri without optic papilledema. JAMA (1972) 220:1591–2. 10.1001/jama.1972.03200120041011 5067736

[3] Marcelis J Silberstein SD. Idiopathic intracranial hypertension without papilledema. Arch Neurol. (1991) 48:392–9. 10.1001/archneur.1991.00530160060014 2012512

[4] Mathew NT Ravishankar K Sanin LC. Coexistence of migraine and idiopathic intracranial hypertension without papilledema. Neurology (1996) 46:1226–30. 10.1212/WNL.46.5.1226 8628457

[5] Wang SJ Silberstein SD Patterson S Joung WB Idiopathic intracranial hypertension without papilledema. A case-control study in a headache centre. Neurology (1998) 51:245–9. 9674810 10.1212/wnl.51.1.245

[6] Bono F Messina D Giliberto C Cristiano D Broussard G Fera F. Bilateral transverse sinus stenosis predicts IIH without papilledema in patients with migraine. Neurology (2006) 67:419–23. 10.1212/01.wnl.0000227892.67354.85 16894101

[7] Bono F Messina D Giliberto C Cristiano D Broussard G D'Asero S. Bilateral transverse sinus stenosis and idiopathic intracranial hypertension without papilledema in chronic tension-type headache. J Neurol. (2008) 255:807–12. 10.1007/s00415-008-0676-2 18458863

[8] Quattrone A Bono F Oliveri RL Gambardella A Pirritano D Labate A. Cerebral venous thrombosis and isolated intracranial hypertension without papilledema in CDH. Neurology (2001) 57:31–6. 10.1212/WNL.57.1.31 11445624

[9] Bono F Quattrone A Idiopathic intracranial hypertension without papilledema in headache sufferers. Cephalalgia (2009) 29:593–4. 10.1111/j.1468-2982.2008.01765_1.x 19170702

[10] De Simone R Ranieri A Montella S Cappabianca P Quarantelli M Esposito F Intracranial pressure in unresponsive chronic migraine. J Neurol. (2014) 261:1364–73. 10.1007/s00415-014-7355-2 PMC4097326 24781838

[11] Fishman RA Cerebrospinal Fluid in Diseases of the Nervous System. 2nd edition Philadelphia, PA: W.B. Saunders Company (1992) p. 71–101.

[12] Bono F Salvino D Tallarico T Cristiano D Condino F Fera F. Abnormal pressure waves in headache sufferers with bilateral transverse sinus stenosis. Cephalalgia (2010) 30:1419–25. 10.1177/0333102410370877 20974602

[13] Spence JD Amacher LA Willis NR. Benign intracranial hypertension without papilledema: role of 24-hour cerebrospinal fluid pressure monitoring in diagnosis and management. Neurosurgery (1980) 7:326–36. 10.1227/00006123-198010000-00004 7442975

[14] Torbey MT Geocadin RG Razumovsky AY Rigamonti D Williams MA. Utility of CSF pressure monitoring to identify idiopathic intracranial hypertension without papilledema in patients with chronic daily headache. Cephalalgia (2004) 24:495–502. 10.1111/j.1468-2982.2004.00688.x 15154860

[15] Galetta SL Digre KB. Misdiagnosing idiopathic intracranial hypertension: you've got some nerve. Neurology (2016) 86:318–9. 10.1212/WNL.0000000000002320 26718571

[16] Lundberg N Continuous recording and control of ventricular fluid pressure in neurosurgical practice. Acta Psychiatr Neurol Scand. (1960) 149(Suppl.): 1–193. 13764297

[17] Fera F Bono F Messina D Gallo O Lanza PL Auteri W. Comparison of different MR venography techniques for detecting transverse sinus stenosis in idiopathic intracranial hypertension. J Neurol. (2005) 252:1021–25. 10.1007/s00415-005-0710-6 15742111

[18] Ueno H Shima K Chigasaki H Ishii S. Oscillation of intracranial pressure and sleep. Clin Neurol Neurosurg. (1986) 88:163–7. 10.1016/S0303-8467(86)80023-3 3096622

[19] Krauss JK Droste DW Bohus M Regel JP Scheremet R Riemann D. The relation of intracranial pressure B-waves to different sleep stages in patients with suspected normal pressure hydrocephalus. Acta Neurochir. (1995) 136:195–203. 10.1007/BF01410626 8748854

[20] Alperin N Sang HL Sivaramakrishnan S Hushek SG. Quantifying the effect of posture on intracranial physiology in humans by MRI flow studies. J Magn Reson Imaging (2005) 22:591–6. 10.1002/jmri.20427 16217773

[21] Bono F Giliberto C Lavano A Quattrone A. Posture-related cough headache and orthostatic drop in lumbar CSF pressure. J Neurol. (2005) 252:237–8. 10.1007/s00415-005-0623-4 15729536

[22] Strassman AM Raymond SA Burstein R. Sensitization of meningeal sensory neurons and the origin of headaches. Nature (1996) 384:560–4. 10.1038/384560a0 8955268

[23] Schankin CJ Ferrari U Reinisch VM Birbaum T Goldbrunner R Straube A Characteristic of brain tumour-associated headache. Cephalalgia (2007) 27:904–11. 10.1111/j.1468-2982.2007.01368.x 17635527

[24] Kirby S Purdy RA. Headache and brain tumors. Curr Neurol Neurosci Rep. (2007) 7:110–6. 10.1007/s11910-007-0005-7 17324360

[25] Taylor LP. Mechanism of brain tumor headache. Headache (2014) 54:772–5. 10.1111/head.12317 24628259

[26] Czosnyka M Pickard JD. Monitoring and interpretation of intracranial pressure. J Neurol Neurosurg Psychiatry (2004)75:813–21. 10.1136/jnnp.2003.033126 15145991 PMC1739058

[27] Newell DW Aaslid R Stoos R Reulen HJ. The relationship of blood flow velocity fluctuations to intracranial pressure B-waves. J Neurosurg. (1992) 76:415–21. 10.3171/jns.1992.76.3.0415 1738020

[28] Risberg J Lundberg N Ingvar DH Regional cerebral blood volume during acute transient rise of the intracranial pressure (plateau waves). J Neurosurg. (1969) 31:303–10. 10.3171/jns.1969.31.3.0303 5811832

[29] Alperin N Lam BL Tain RW Ranganathan S Letzing M Bloom M. Evidence for altered spinal canal compliance and cerebral venous drainage in untreated idiopathic intracranial hypertension. Acta Neurochir. Suppl. (2012) 114:201–5. 10.1007/978-3-7091-0956-4_39 22327693

[30] Bono F Lupo MR Serra P Cantafio C Lucisano A Lavano A Obesity does not induce abnormal CSF pressure in subjects with normal MR venography. Neurology (2002) 59:1641–43. 10.1212/01.WNL.0000035628.81384.5F 12451215

[31] Whiteley W Al-Shahi R Warlow CP Zeidler M Lueck CJ. CSF opening pressure: reference interval and the effect of body mass index. Neurology (2006) 67:1690–1. 10.1212/01.wnl.0000242704.60275.e9 17101909

[32] Bono F Cristiano D Mastrandrea C Latorre V D'Asero S Salvino D. The upper limit of normal CSF opening pressure is related to bilateral transverse sinus stenosis in headache sufferers. Cephalalgia (2009) 30:145–51. 10.1111/j.1468-2982.2009.01896.x 19515130

[33] Bono F Lupo MR Lavano A Mangone L Fera F Pardatscher K. Cerebral MR venography of transverse sinuses in subjects with normal CSF pressure. Neurology (2003) 61:1267–70. 10.1212/01.WNL.0000092021.88299.B4 14610135

[34] Fisayo A Bruce BB Newman NJ Biousse V. Overdiagnosis of idiopathic intracranial hypertension. Neurology (2016) 86:341–50. 10.1212/WNL.0000000000002318 26718577 PMC4776085

