# Supplementary material for: Cerebrospinal Fluid Pressure-Related Features in Chronic Headache: A Prospective Study and Potential Diagnostic Implications
Source: Front Neurol. 2018 Dec 18;9:1090. doi: 10.3389/fneur.2018.01090 (PMC6305580; doi:10.3389/fneur.2018.01090)
Supplement: Supplementary file 1 [file Table_1.pdf]

**Supplementary Table 1 – Post-hoc comparisons**

| *Pairwise comparisons with Bonferroni correction* | Group 1 vs Group 2<br>*p value* | Group 1 vs. Group 3<br>*p value* | Group 2 vs Group 3<br>*p value* |
|---|---|---|---|
| CSF pressure measurement | | | |
| Pressure pulsations | <0.001 | <0.001 | 1 |
| Headache diagnosis | | | |
| Pre-existing primary headache | <0.001 | <0.001 | 1 |
| Headache profile | | | |
| Pulsating pain | 0.051 | <0.001 | 1 |
| Severe | <0.001 | <0.001 | <0.001 |
| Daily | <0.001 | <0.001 | 0.005 |
| Aggravated with coughing | <0.001 | <0.001 | 0.01 |
| Nocturnal head pain attacks | <0.001 | <0.001 | 0.046 |
| Positional headache | <0.001 | <0.001 | 0.64 |
| Associated symptoms | | | |
| Pulsatile Tinnitus | 0.01 | <0.001 | 1 |
| Visual disturbances | 0.006 | <0.001 | 0.001 |
| Intracranial noises | 0.005 | <0.001 | 0.09 |
| Neuroimaging findings | | | |
| Empty sella | <0.001 | <0.001 | 1 |
| Perioptic subaracnoid space distension | 0.001 | <0.001 | 0.02 |
| Bilateral TSS | <0.001 | <0.001 | 1 |
| Normal-appearing TS | <0.001 | <0.001 | 0.45 |
