# Supplementary material for: Cerebrospinal Fluid Pressure-Related Features in Chronic Headache: A Prospective Study and Potential Diagnostic Implications
Source: Front Neurol. 2018 Dec 18;9:1090. doi: 10.3389/fneur.2018.01090 (PMC6305580; doi:10.3389/fneur.2018.01090)
Supplement: Supplementary file 2 [file Table_2.pdf]

Supplementary Table 2 – Post-hoc comparisons with control group

Pairwise comparisons with Bonferroni correction	Ctrl vs Group 1<br>*p value*	Ctrl vs Group 2<br>*p value*	Ctrl vs Group 3<br>*p value*	Group 1 vs Group 2<br>*p value*	Group 1 vs. Group 3<br>*p value*	Group 2 vs Group 3<br>*p value*
BMI	1	0.038	<0.001	<0.001	<0.001	1
CSF pressure measurement						
Opening pressure	0.1	<0.001	<0.001	<0.001	<0.001	<0.001
Mean pressure	1	<0.001	<0.001	<0.001	<0.001	<0.001
Highest Peak pressure	1	<0.001	<0.001	<0.001	<0.001	<0.001
Pulse amplitude	<0.001	<0.001	<0.001	<0.001	<0.001	<0.001
