# Supplementary material for: Cerebrospinal Fluid Pressure-Related Features in Chronic Headache: A Prospective Study and Potential Diagnostic Implications
Source: Front Neurol. 2018 Dec 18;9:1090. doi: 10.3389/fneur.2018.01090 (PMC6305580; doi:10.3389/fneur.2018.01090)
Supplement: Supplementary file 3 [file Table_3.pdf]

Supplementary Table 3 – Generalized Linear Models

Outcome	*Effect of*	*coefficient (exp)*	*confidence interval (exp)*	*p value*
Pressure pulsations	pulse amplitude	1.033	1.023 – 1.045	<0.001
Nocturnal head pain attacks	fluctuations	6.33	2.71 – 16.24	<0.001
Postural variations	fluctuations	17.56	3.27 – 365.55	0.007
Photo/phono phobia	fluctuations	2.61	1.34 – 5.15	0.005
Pulsatile tinnitus	fluctuations	5.65	2.8 – 11.8	<0.001
Visual disturbances	fluctuations	37.35	4.9 – 411.1	<0.001
	pulse amplitude	1.03	1.01 – 1.05	0.0035
	fluctuations: pulse amplitude	0.98	0.956 – 0.997	0.029
vertigo	pulse amplitude	1.01	1.0 – 1.02	0.008
